# The Facial Skeleton in Patients with Osteoporosis: A Field for Disease Signs and Treatment Complications

**DOI:** 10.4061/2011/147689

**Published:** 2011-02-16

**Authors:** Athanassios Kyrgidis, Thrasivoulos-George Tzellos, Konstantinos Toulis, Konstantinos Antoniades

**Affiliations:** ^1^Department of Oral and Maxillofacial Surgery, Faculty of Dentistry, Aristotle University of Thessaloniki, Thessaloniki 54124, Greece; ^2^Department of Pharmacology, Faculty of Medicine, Aristotle University of Thessaloniki, Thessaloniki 54124, Greece; ^3^Department of Endocrinology, 424 Military Hospital, Thessaloniki 56429, Greece

## Abstract

Osteoporosis affects all bones, including those of the facial skeleton. To date the facial bones have not drawn much attention due to the minimal probability of morbid fractures. Hearing and dentition loss due to osteoporosis has been reported. New research findings suggest that radiologic examination of the facial skeleton can be a cost-effective adjunct to complement the early diagnosis and the follow up of osteoporosis patients. Bone-mass preservation treatments have been associated with osteomyelitis of the jawbones, a condition commonly described as osteonecrosis of the jaws (ONJ). The facial skeleton, where alimentary tract mucosa attaches directly to periosteum and teeth which lie in their sockets of alveolar bone, is an area unique for the early detection of osteoporosis but also for the prevention of treatment-associated complications. We review facial bone involvement in patients with osteoporosis and we present data that make the multidisciplinary approach of these patients more appealing for both practitioners and dentists. With regard to ONJ, a tabular summary with currently available evidence is provided to facilitate multidisciplinary practice coordination for the treatment of patients receiving bisphosphonates.

## 1. Introduction

Osteoporosis is a very common medical condition affecting over 5% of the global population [1, 2]. A considerable proportion of these patients will sustain one or more fragility fractures in their remaining lifetime [1–3]. Despite its low case fatality, morbidity from osteoporosis poses important socioeconomic burden [4]. The incidence of osteoporosis has been known to be on the rise; however, a break in this trend has been reported in the last decade [5]. Therefore, osteoporosis is considered as a serious public health concern which affects both genders [1, 6]. Expectedly, research is focused in the development of new treatments for osteoporosis while a variety of drugs have been made available during the past 50 years. The treatment of osteoporosis involves management of osteoporosis-associated fractures, universal prevention measures, and medical treatment of the underlying disease. Orthopaedic surgeons deal with osteoporotic fractures, while the nutrition and exercise are the leading prevention measures to reduce risk factors for osteoporosis. Medications are recommended for patients at high risk of fractures to reduce fractures. Risk factors to be considered in making the treatment decision include age, prior history of fracture, family history of fracture, weight, underlying diseases and medications, bone mineral density, and current smoking [7]. Currently approved medications include alendronate (Fosamax), risedronate (Actonel), and raloxifene (Evista) for prevention and treatment of osteoporosis; teriparatide (Forsteo), denosumab (Prolia), zolendronic acid (Aclasta), ibandronate (Bondenza) nasal calcitonin spray (Miacalcin) for treatment only, and estrogens or combinations of hormones (hormone replacement therapy (HRT)) for prevention only. Osteoporosis is asymptomatic until a fracture occurs, which poses a major challenge for the treating physician and may in part explain why relatively few patients receive a diagnosis of osteoporosis [8]. Bone mineral density (BMD) has been reported to correlate for more than three quartiles of total bone strength [9, 10]. Quantization of BMD to predict the risk of fractures is of the same order of importance and efficacy as measuring blood pressure or cholesterol levels to predict the risk of experiencing stroke or myocardial infarction [8–10]. Spine and hip BMD signify the risks of experiencing vertebral and hip fractures. The gold standard method for determining BMD is dual energy X-ray absorptiometry (DXA). Other BMD measures include peripheral DXA, calcaneal ultrasonography [11] and digital X-ray radiogrammetry, and they are used to screen for and to predict the short-term risk of experiencing fracture [12–14]. The World Health Organization (WHO) defines osteoporosis in postmenopausal women as a BMD with T score over 2.5 standard deviations below the mean for young healthy adults. A BMD between 1.0 and 2.5 standard deviations below the mean (*T* score = −1.0 to −2.5) is classified as osteopenia. The risk of fracture is proportionate to the decrease in BMD. Nonetheless, more fractures are seen in people with osteopenia than in people with osteoporosis, because the number of people with osteopenia is higher [9, 10, 14–16]. Personal risk factors for hip fracture [15, 16] have been identified from epidemiologic studies such as the Study of Osteoporotic Fractures [8, 15].

The facial skeleton is different from the remainder of skeleton; the main difference is the fact that it comprises membranous rather than chondrogenous bones. Bone development occurs in two main forms. The majority of bones is preformed in cartilage which is later replaced (endochondral ossification, endochondral bone). However, in the skull and the clavicle, bone forms directly in membranous connective tissue (intramembranous ossification, membranus bone). A brief look at the history of the skeleton may explain why [17, 18]. Calcified skeletal tissues replaced silicacious in the Cambrian period, most probably because physiological changes either in the beasts or the oceans in which they lived, allowed retention of Ca ions. It was then those brachiopods, nautiloids, trilobites gradually converted. Later the first vertebrates had bony scales embedded in their skin—those around the mouth incidentally forming the primitive basis of teeth. In some phylogeny lineages, these scales fused to form bony carapaces [17, 18]. Humans retained these carapaces over our heads as skull vaults. Later the rest of the skeleton like the vertebrae, which were cartilaginous also became bony. This phylogeny explains the distribution and origins of membrane and cartilaginous bone. Facial bones directly ossify from mesenchyme. The surviving membranous bones in the head and part of the clavicle are fragments of the dermal shield. The formation of membranous bone from neural crest-derived mesenchyme of the maxillary and mandibular processes of the embryo depends upon preceding interactions between the mesenchyme and maxillary or mandibular epithelia. These epithelial-mesenchyme interactions that initiate osteogenesis in both the mandibular and the maxillary processes have been reported to be permissive interactions [19]. Centres of ossification that are marked by the appearance of calcified matrix appear during lifetime, some in embryonic life, others in fetal and yet others well into the postnatal growing period. Some bones ossify from a single ossification centre, others from a group, of which one is the primary (central and premature) and the remainder secondary (later and often peripheral) [17]. The bones in the skull and facial complex remain separated by a fibrous union (suture) until the seventh or eighth decade of life [20]. Sutures function as intramembranous bone growth sites that remain in an unossified state, to allow new bone to be formed at the edges of the overlapping bone fronts. This process relies on the production of sufficient new bone cells which are recruited into the bone fronts, while ensuring that the cells within the suture remain undifferentiated. Contrary to endochondral growth plates that can expand through chondrocyte hypertrophy, sutures do not posses intrinsic growth potential. Rather, they produce new bone at the sutural edges of the bone fronts in response to external stimuli, such as signals arising from the expanding neurocranium or from facial muscles tension [20]. 

Phylogeny which was primarily based on observations is nowadays discovered through molecular sequencing data and morphological data matrices [18, 21]. Depending on their membranous or endochondral origin, bones have distinct signaling properties, which need to be considered in the research and application of bone biology [22, 23]. This is not theoretical as clinical implications have already been reported [24]. In this regard, we review issues related to the facial skeleton in patients with osteoporosis. 

## 2. Inner Ear Issues in Patients with Osteoporosis

Hearing loss in patients with osteoporosis has long been described. The majority of reported osteoporotic patients with hearing loss were affected by Paget disease [25]. The hormonal control of bone metabolism has taken on a new dimension since the description, within the last decade, of a major osteoclast inhibiting control system. The receptor activator of nuclear factor-[kappa]B (NF-[kappa]B) ligand (RANKL) produced by osteoblastic lineage cells, binds with its receptor RANK, located on osteoclasts, in order to allow the maturation and activation of osteoclasts [26]. The potential continuous bone loss is controlled by the decoy receptor osteoprotegerin (OPG) which competitively binds to RANKL and hence blocks the interaction of RANKL-RANK [26, 27]. Estrogens contribute to bone protection since they decrease the response of osteoclasts to RANKL and induce osteoclast apoptosis. But estrogens, are stimulators of prolactin release. Prolactin affects calcium metabolism and pregnancy-induced hyperprolactinemia affects BMD. Long-term estrogen treatment in guinea pig results in hyperprolactinemia and has been reported to lead to hearing loss as well as bone dysmorphology of the otic capsule [28]. Recent data show that prolactin decreases OPG and increases RANKL [29]. OPG has been shown to be expressed at high levels in the cochlea and OPG knock-out mice have indeed abnormal remodeling of the otic capsule and resorption of the auditory ossicles [30]. This might explain why oral contraception treatment and hormone replacement therapies, involving estrogen together with progestin, increase the risk of otosclerosis and vestibular disorders [31]. Benign paroxysmal positional vertigo (BPPV), also called canalolithiasis and cupulolithiasis has been associated with lower T-scores in postmenopausal women. The diagnosis of osteopenia or osteoporosis was confirmed by a bone mineral density measurement made with DXA of spine and hip (T-score) [32]. These results suggested a possible relationship between recurrent BPPV and a decreased fixation of calcium in bone in postmenopausal women. In an experimental model used to test this hypothesis, in which osteopenia/osteoporosis was induced by bilateral ovariectomy in female rats, the density of otoconia was decreased and their size was increased when compared to the control group. Utricular otoconia of both groups of rats examined by conventional and epifluorescence microscopy, labeling with calcein showed lack of external calcium turnover into otoconia of adult female rats [33]. 

## 3. Oral Health in Patients with Osteoporosis

Several other factors also affect the dental management of this disease. Patients diagnosed with or at high risk for developing osteoporosis often have other chronic diseases. These patients' oral health is also compromised since they are receiving medications to treat these diseases and due to physical disability and poor hygiene compliance issues. They usually have major dental requirements and their poor oral health can cause systemic health deterioration. Preserving the natural dentition of those patients promotes better nutrition and improves appearance [34, 35]. On the other hand, poor oral health in this population can contribute to increased morbidity and decreased quality of life [35–37]. People with chronic diseases and poor oral health are at increased risk of developing opportunistic infections such as pneumonia and of xerostomia induced by medications [35, 36]. Patients' poor oral hygiene, loss of hand dexterity, lack of compliance and poor dentition can, in turn, impair oral function [35, 38]. Therefore, dental care is indicated for these patients; to provide satisfactory care, dentists need to understand osteoporosis, its treatments and its complications. A number of review articles about osteoporosis and periodontal disease discussed various issues regarding BMD and oral alveolar bone loss, premature teeth loss and increased severity of periodontal disease in patients with osteoporosis [39–41]. Common risk factors for osteoporosis and periodontal disease include smoking, old age, and low intake of calcium and vitamin D [8]. Since both osteoporosis and periodontitis are highly prevalent and markedly associated with aging, studies have been performed to investigate the association between these diseases over the past decades [39, 40, 42]. Experimental results [43] suggest that despite those studies, no clear association between these diseases exist other than common risk factors. Through recognizing common risk factors for both osteoporosis and periodontal disease and performing clinical and radiographic dental examinations dentists identify patients who are at risk of developing osteoporosis. The results of radiographic assessment of the alveolar trabecular pattern can be a clinical indicator of BMD [44]. Other studies suggest that routine panoramic radiographs also can be used to detect low BMD, osteoporosis and risk of experiencing vertebral fracture in postmenopausal women [45–48]. These studies also showed that providing special training to dental practitioners on specific evaluation techniques and reading panoramic radiographs enhanced their detection of osteoporosis related radiographic changes. Briefly, the radiographic examination of the mandibular inferior cortex can reveal changes that vary from normal with the endosteal cortical margins being even and sharp bilaterally, to mild or moderate erosion of the inferior cortex, to severe erosion and presence of heavy endosteal cortical residues and porosity of the inferior mandibular cortex, unilaterally or bilaterally. Panoramic X-rays are cheap and routinely performed in many patients, in contrast with DXA which may be too expensive to be widely implemented in population screening programs. Some authors concluded that panoramic X-rays can help detect a high percentage of postmenopausal women with undetected low BMD, as well as undetected spinal fractures which may then be referred for DXA [45–52]. Under the auspices of a European Union Initiative (the OSTEODENT project), a special computer software has been developed to facilitate early diagnosis of osteoporosis by dental practitioners. The cost-effectiveness of the program has been documented [51–58]. Physicians and dentists have a shared interest to identify patients at risk of developing osteoporosis and periodontal disease. Collaboration between these professionals to early diagnose osteoporosis and osteopenia can lead to early osteoporosis therapy and prevention of fractures. 

## 4. Osteonecrosis of the Jaws (ONJ)

Osteonecrosis of the jaws (ONJ) was initially described as an oral complication resulting from undergoing bisphosphonate therapy and is to date defined as the presence of necrotic bone anywhere in the oral cavity in a patient who is taking a bisphosphonate, who has not received radiation to the head and neck and in whom the necrotic area does not heal within eight weeks after diagnosis after receiving proper care [7, 59, 60]. Patients with ONJ were classified in three stages, while in 2009 a stage 0 was also proposed [61] and subsequently adopted (Table 1) [59, 62]. Most reported cases of ONJ have been associated with the intravenous administration of zoledronic acid or pamidronate in patients with cancer-related conditions, including bone metastases in the context of solid tumors such as breast cancer, prostate cancer, and lung cancer, and lytic lesions in the setting of multiple myeloma [62–64]. ONJ also has been diagnosed, although in a smaller number, in patients taking oral bisphosphonates such as alendronate, risedronate, ibandronate and clodronate for the prevention and treatment of osteoporosis [65–67]. Various aetiopathogenetic paradigms have been proposed.  Table 2 briefly summarizes those most plausible. Predisposing factors that have been proposed to be associated with the development of ONJ in patients under BP treatment include dental extractions [68–70], use of dentures [68, 69], presence of periodontal disease [69, 71], smoking [68, 69, 72], diabetes mellitus [62], glucocorticoid use [62] and prolonged bisphosphonate therapy [69, 73]. Thus, reports of ONJ have implications for the dental care of patients with osteoporosis [65]. It is important for dental practitioners to identify patients who are taking a bisphosphonate. Due to the fact that the majority of bisphosphonates are administred either weekly or monthly, patients frequently forget to disclose to dentists that they are taking the medication. Including specific questions about osteoporosis and bisphosphonate use in the dental history may facilitate the identification of those under BP treatment.

The ideal dental management protocol for patients taking oral bisphosphonates has been a matter of debate. It has been suggested that patients need be given a “drug holiday” when surgical dental intervention that includes bone manipulation is scheduled [91]. Existing evidence; however, provides no scientific grounds to support the theory that discontinuation of bisphosphonate therapy will improve treatment outcomes [67, 92]. Therefore, before discontinuing bisphosphonate therapy, dentists and physicians must collaborate to determine the best way to manage the treatment of each patient. Several health indicators, including BMD, degree of risk of experiencing spine and hip fractures and duration of bisphosphonate therapy would need to be discussed in such a consultation. This consultation also would help health care practitioners make a decision about whether a drug holiday is acceptable for any individual [8]. 

The risk of fracture following treatment over a period of time and subsequent discontinuation of an oral bisphosphonate for patients with osteoporosis has not been well established. The Fracture Intervention Trial Long-term Extension (FLEX) evaluated the effects of continuing or stopping oral alendronate in postmenopausal women for up to 10 years. Investigators found that BMD was maintained and bone remodeling was suppressed with no detectable increase in fracture risk [93]. In the group of women who discontinued oral alendronate use after five years, the BMD and bone remodeling were maintained at higher levels than those obtained at baseline. The BMD and bone marker changes suggested some residual effect from 5 years of alendronate treatment that is evident for at least 5 years after discontinuation [93]. 

The association of hip fracture with high mortality also is important, however the potential savings from hip fracture prophylaxis may be overestimated by studies that fail to consider differential risk, mortality, and long-term followup [94]. Managing the care of a patient who has ONJ and is taking a bisphosphonates is based mostly on expert opinion [95]. Several strategies have been attempted, including sequestrectomy, surgical local debridement and periodontal flap surgery, as well as less invasive procedures like antibiotic therapy and mouthrinses [67, 78, 86, 95, 96]. Treatment outcomes vary from complete healing to partial healing to no healing. Both practitioners and dentists must keep in mind that the management of ONJ is difficult and no definite treatment exists to date. The osteonecrotic process usually does not respond to routine therapy, and more aggressive surgical manipulation of the area is not recommended [97]. In those ONJ cases when there is clinical evidence of active infection, conservative approaches such as minor local debridement and systemic antibiotic therapy are indicated. When there is trauma to the soft tissues sharp bone edges should be eliminated. Routine oral hygiene maintenance is indicated, and it can be complemented with topical chlorhexidine rinses [59, 62, 67, 97]. Table 1 summarizes proposed interventions for patients receiving BPs but also for those who developed ONJ.

When a patient taking oral bisphosphonate needs to undergo a surgical procedure, Marx et al. suggested that the patient discontinue BPs and undergo a serum C-telopeptide test of type I collagen (CTX) prior to the procedure [98]. CTX is used to measure bone resorption and detect the fragments of collagen type I peptide released in the circulatory system when osteoclasts resorb bone. The authors recommended that when the CTX level is higher than 150 picograms per milliliter, the risk of developing ONJ following an invasive dental surgical procedure is diminished [98]. There are, however, no sufficient data to support the use of CTX to predict the development of ONJ. An expert panel recommended that using this test may not be an evidence-based approach as a control group was missing in the initial study [99]. Other authors also commented on the lack of quality evidence with regard to the predictive value of CTX [100] while a study with limited followup concluded that serum CTX is not a valid preoperative test to accurately assess the level of risk of developing ONJ and is not indicated in the oral surgery patient [101]. Because ONJ can be a devastating complication of bisphosphonate therapy, a collaborative effort between dentists and physicians in deciding on the patient's dental treatment is recommended, because it can minimize complications and adverse events. The reported incidence of ONJ in patients taking oral bisphosphonates is relatively low, which may be due to underreporting, different duration of therapy in countries that have adopted bisphosphonates more recently and different definitions of ONJ [65, 66]. There are an estimated 0.7 cases per 100,000 patient-treatment years in the United States [74, 102]. However, some geographic variations in incidence are being reported such as in Australia, where the number of cases could be much higher [103]. Others believe that the incidence of ONJ is low, considering the millions of patients with osteoporosis who are taking oral bisphosphonates [67]. The development of new bisphosphonates may enhance the safety of this medication. A recent clinical trial reported the results of treating patients with osteoporosis with a new formulation of bisphosphonate (Zoledronic acid 5 mg, Aclasta) [104]. Patients received an annual intravenous infusion of 5 milligrams of zoledronic acid for a period of three years. The trial demonstrated a significant reduction of the risk of vertebral, hip and other fractures. Only two cases of ONJ were detected; one in the treatment group, and one in the placebo group [104]. It is not uncommon, however, that drug adverse events emerge only after the drug receives US Food and Drug Administration approval on a postmarket basis and is widely adopted in everyday clinical use [7]. 

## 5. Specific Osteoporosis Treatment Agents

Denosumab is a human monoclonal IgG2 antibody that binds selectively and with high affinity to the receptor activator of nuclear factor-*κ*B ligand (RANKL) and pharmacologically mimics the effect of osteoprotegerin on RANKL [26, 27, 88] thereby blocking the binding of RANKL to the receptor activator of nuclear factor-*κ*B (RANK). Denosumab rapidly decreases bone turnover markers resulting in a significant increase in bone mineral density and reduction in fracture risk [26, 27, 88]. Amgen's denosumab was approved under the brand name Prolia for osteoporosis in mid-2010 [105]. The safety and efficacy of Prolia in the treatment of postmenopausal osteoporosis was demonstrated in a three-year, randomized, double-blind, placebo-controlled trial of 7,808 postmenopausal women ages 60 to 91 years. In the study, Prolia reduced the incidence of vertebral, nonvertebral, and hip fractures in postmenopausal women with osteoporosis [106]. Of note, in the latter study previous bisphosphonate administration was a possible confounder; however, the issue has been addressed by the authors [107]. Recently, denosumab was granted with FDA approval for the prevention of skeletal-related events in patients with bone metastases from solid tumors under the trade name Xgeva [108]. While ONJ incidence with denosumab in clinical trials has been negligible in those patients with osteoporosis, in metastatic cancer patients ONJ has been recorded as an adverse effect [88, 109]. Importantly, it has been suggested that since denosumab exhibits the advantage of short clearance time when compared to bisphosphonates, more feasible treatment and earlier healing of denosumab-related ONJ when compared to bisphosphonate-related ONJ could be anticipated [88].

Teriparatide, which consists of the N-terminal 34 amino acids of parathyroid hormone, has been in clinical use for the treatment of osteoporosis for almost a decade, ever since clinical trials showed that among patients with severe osteoporosis who were treated with teriparatide, the relative risks of vertebral and nonvertebral fractures were reduced by 65% and 53%, respectively [109, 110]. Unlike bisphosphonates, the current first-line agents for the prevention of fractures, which act primarily by inhibiting bone resorption, teriparatide increases bone density and strength primarily by stimulating osteoblastic bone formation. Thus, teriparatide stimulates bone remodeling, whereas bisphosphonates decrease it [109]. A recent study reported improved clinical outcomes, greater resolution of alveolar bone defects, and accelerated osseous wound healing in a yearly followup in the oral cavity of patients with chronic periodontitis who underwent periodontal surgery and received daily injections of teriparatide or placebo, along with oral calcium and vitamin D supplementation, for 6 weeks [110]. Teriparatide may offer therapeutic potential for localized bone defects in the jaw. Furthermore, teriparatide has been reported to promote the spontaneous resolution of ONJ. Despite the fact that the only three cases have been published to date [111–114], given the FDA approval of teriparatide for osteoporosis and the limited existing evidence with regard to ONJ healing, it could be justified to prescribe teriparatide to patients with bisphosphonate-treated osteoporosis who already have ONJ. 

## 6. Conclusions

The facial skeleton is a region of particular interest in patients with osteoporosis. Firstly, inner ear pathophysiology and manifestations may be related to calcium metabolism. Evidence suggests that sex hormones convey changes to the otoconia of the cochlea and the vestibule. Postmenopausal osteoporosis is known to be associated with sex hormone changes, and may be associated with benign paroxysmal positional vertigo. Practitioners should be aware of these symptoms and early refer their patients to ENT surgeons. Secondly, although the relation between osteoporosis and periodontal disease has not been quantified, maintenance of optimal oral hygiene would likely be beneficial for osteoporosis patients. Furthermore, panoramic X-rays widely used in dentistry are of importance to early refer selected patients for DXA screening. Good knowledge of osteoporosis specific alterations in panoramic X-rays is a prerequisite and dentists should be keen on referring these patients. Thirdly, osteonecrosis of the jaws is one of the most discussed complications resulting from bone mass preservation treatment. Prevention and timely diagnosis of this complication requires awareness and collaboration from both physicians and dentists. Similar to the paradigm of bisphosphonates and ONJ, the broad introduction of denosumab and teriparatide might bear skeletal-related complications but it might also introduce new therapeutic potentials. Early recognition of future complications and early exploitation of therapeutic potentials mandate for a multidisciplinary approach. 

## Figures and Tables

**Table 1:** Staging classification of osteonecrosis of the jaws by bisphosphonates and treatment strategies (from Kyrgidis et al [59], modified on the basis of current evidence).

Osteonecrosis of the jawsstaging	Stage description	Treatment strategies
Future risk category	Candidate patients to be enrolled in bisphosphonate treatment, patients who have enrolled to bisphosphonate treatment for a period shorter than 3 months	“*Patient education*” (inform patients about the complication, its signs, and symptoms) [74]
		*“Maintain optimal oral hygiene”* (biannual periodontal scaling, restoration decayed teeth) [63, 74, 75]
		*“Provide root canal treatment as usual”* [68, 69]
		*“Treat active oral infections, remove sites at high risk for infection”* (partially impacted wisdom teeth, nonrestorable teeth, teeth with extensive periodontal dehiscence) [60, 74, 75]
		*“Check for ill-fitting dentures, retread if necessary”* [68, 69]
		Baseline dental evaluation (history taking, clinical examination and panoramic radiographs) [68, 74, 75]

At-risk category	No apparent necrotic bone in patients who have been treated with either oral or IV bisphosphonates	All of the above
		*“Prefer conservative dental treatment modalities over dental extractions”* (root canal treatment, periodontal scaling and root planning) [68–70, 74]
		Perform extractions and other surgery only when utterly inevitable; in such cases use minimal bone manipulation with appropriate local and systemic antibiotics [68–70, 76]:
		(i) Perform periodontal scaling 3 weeks prior
		(ii) Prescribe amoxicillin 1gr t.i.d. 3 days prior
		(iii) Reflect full thickness mucoperiosteal flap, remove teeth with minimal cortical trauma
		(iv) Suture and prescribe amocicillin 1 gr t.i.d. for 17 days, chlorexidine 1% rinses t.i.d.
		(vi) Remove sutures and discontinue chlorexidine rinses 1 week postoperatively
		(v) Prefer single tooth interventions
		(vi) Followup to ensure healing

Stage 0	No clinical evidence of necrotic bone, but non-specific clinical findings and symptoms	All of the above
		*“Systemic management”*, including use of pain medication and antibiotics [62]

Stage 1 [77]	Exposed bone necrosis or small oral ulceration without exposed bone necrosis, but without symptoms [77]	All of the above
		*“Oral antibacterial mouth rinse”* (0.12% chlorhexidine rinse, hydrogen peroxide)
		*“Impede denture use”* [68, 69]
		*Discontinuation of bisphosphonate therapy until osteonecrosis heals or underlying disease progresses is not indicated but might be individually considered prior to surgery* [62, 78–80]
		Clinical followup on quarterly basis [62]

Stage 2a [77]	Exposed bone necrosis or a small oral fistula without exposed bone necrosis, but with symptoms controlled with medical treatment [77]	All of the above
		Suggest computed tomography scans
		*Symptomatic treatment* with oral antibiotics (monotherapy or combination therapy with b-lactam, tetracycline, macrolide, metronidazole, or clindamycin) [74]
		*“Pain control”* with non-steroid anti-inflammatory drugs

Stage 2b [77]	Exposed bone necrosis or a small oral fistula without exposed bone necrosis, but with symptoms not controlled with medical treatment [77]	All of the above
		Superfcial debridement to relieve soft tissue irritation

Stage 3 [77]	Jaw fractures, skin fistula, osteolysis extending to the inferior border [77]	All of the above
		Surgical debridement/resection for longer term palliation of infection and pain under intravenous antibiotic treatment
		Use of doxycycline bone fluorescence to discriminate viable bone [81, 82]

**Table 2:** Most plausible aetipathogenetic paradigms for osteonecrosis of the jaws (ONJ).

Paradigm	Synopsis	Citations
Osteoclast-mediated toxicity	Bisphosphonates suppress osteoclast-mediated bone remodeling. This suppression results in “fatigue” of the alveolar bone, responsible for necrosis	[83–85]

Soft tissue toxicity	The oral mucosa is initially involved. As the damage progresses, underlying alveolar bone is also involved and the clinical presentation of ONJ becomes evident	[79, 86]

Infection	Increased bacterial adhesion to the bisphosphonate covered bone may be the cause for ONJ development	[84, 87]

Impaired immune homeostasis-macrophage impaired function	Dendritic cells, macrophages, cytotoxic and helper T-lymphocytes are affected by bisphosphonates. Chemokines, like tumor necrosis factor-alpha, inteleukins IL-1a, IL-1b, IL-6 and IL-8 are also impaired by bisphosphonates. Impaired immune response is responsible for continued inflammation resulting in osteomyelitis. Impaired function of macrophages due to RANKL inhibition is a key phenomenon in the defective topical immune response	[79, 88–90]
